# Special Issue: “New Trends in Membrane Preparation and Applications”

**DOI:** 10.3390/molecules25051132

**Published:** 2020-03-03

**Authors:** Francesco Galiano, Alberto Figoli

**Affiliations:** Institute on Membrane Technology (CNR-ITM), Via P. Bucci 17/c, 87036 Rende (CS), Italy

**Keywords:** polymeric membranes, mixed matrix membranes, membrane preparation, membrane characterization, water and wastewater treatment, gas and vapor separation, new solvents in membrane preparation, new polymers in membrane preparation

This Special Issue aims to provide a collection of recent advancements in the field of membrane science. It includes two original research articles, one communication and three review papers. Stringent current topics were discussed covering new approaches for sustainable membrane fabrication, emerging membrane applications in environmental remediation and innovative techniques integrating nanotechnologies and membrane processes.

During the last years, the scientific research in the membrane technology field, has greatly focused his attention on developing more sustainable processes in membrane fabrication trough the replacement of traditional toxic solvents with safer and green alternatives [1,2,3] or through the use of biopolymers with the aim of decreasing the environmental impact connected to the use of fossil-based polymers [4]. In this regard, three contributes, focusing on the preparation of new membranes using more sustainable approaches, have been published.

Huertas et al. [5] prepared ceramic membranes with photocatalytic activity employing a new environmentally-friendly fabrication route. Photocatalytic surfaces were, in fact, produced under a mild temperature (80 °C) and solvent-free conditions (water) and tested for the photodegradation of methylene blue. Silicon carbamide flat sheet membranes acted as substrates for the deposition of TiO2 nanoparticles using the drop-casting method and using water as a matrix. A comparison, in terms of photocatalytic activity, between the new sustainable developed membranes and the membranes prepared with solvent and at high temperatures, was carried out. The membranes prepared with the new approach presented the highest total methylene blue removal equal to 77% (1.6 times higher than the control) and the lowest dye adsorption (17%). The efficiency of this membrane was still maintained after five consecutive photodegradation runs and 160 min was identified as sufficient time for achieving methylene blue degradation under the detection limit. The membranes displayed also good chemical resistance after cleaning or when exposed to acid and basic solutions where no release of TiO2 was observed. This work proved the possibility of preparing photocatalytic ceramic membranes using a more environmentally-friendly process without compromising membrane performance. 

Vera et al. [6] developed a new eco-friendly procedure for the preparation of polymer inclusion membranes (PIMs) avoiding the use of organic harmful solvents. Two cellulose derivatives, polyurethane (PU) and ε-caprolactone (PCL) were selected as eco-friendly polymers and Aliquat 336 was employed as ionic liquid (IL). The PIMs were prepared by the thermal-compression technique avoiding the use of any organic solvent. Membranes prepared with cellulose derivatives suffered from lack of mechanical resistance, thermal degradation and unsuccessful IL entrapment. However, PU and PCL based membranes displayed good mechanical properties and successful IL incorporation. One of the major bottlenecks of PIMs is their tendency to release the IL, over time, when in contact with liquid solutions. Both PIMs prepared in this work, on the contrary, when in contact with water, presented just a slight loss in IL (about 7%), a value much lower than the analogs PIMs prepared with conventional solvent casting methods. Both membranes were found to be efficient for the removal of Cr(VI) anions reaching an extraction efficiency of more than 60% after just 15 min. For the first time, in this work, PIMs based on biodegradable polymers were produced using a solvent-free approach. The prepared membranes displayed outstanding stability and good extraction efficiency.

Biopolymers were the objective of the review of Castro-Muñoz and González-Valdez [7]. The authors provided a state-of-the-art of biopolymers derived from different natural sources (animal, vegetal and microbial fermentation) for the preparation of membranes applied in pervaporation (PV). PV is a membrane process where dense membranes are employed for the separation of liquid mixtures mainly represented by close-boiling azeotropic solutions. The active top layer of the membrane is in contact with the liquid feed solution to be separated while the permeate side, enriched with the target permeating species, is collected as a vapor phase. Most of the biopolymer-based membranes, due to their hydrophilic nature, are applied for the dehydration of organic solvents such as the separation of water from alcohols (ethanol, butanol, isopropanol), from ethylene glycol, dimethyl carbonate and acetone. Some of them find application also in the separation of organic/organic mixtures such as methanol/methyl tert-butyl ether and benzene/cyclohexane. Chitosan, polylactic acid (PLA) and sodium alginate were the most studied biopolymers in PV. Biopolymer-based membranes can suffer of poor mechanical properties. In this regard, the incorporation of inorganic fillers (zeolites, MOF, nanotubes, graphene oxide, etc.) into the polymeric matrix was found to be effective for the improvement of membrane mechanical resistance. 

Two contributes of this Special Issue reviewed the use of membranes in two trending topics in the environmental pollution remediation: microplastics removal from water and CO_2_ capture from flue gas.

The review by Poerio et al. [8] summarised the crucial role that membranes can have in reducing the impact of plastic pollution in the aquatic environment. Water contamination from plastics is a worldwide urgent issue still waiting for a concrete and efficient solution. Membrane separation techniques, in this regard, have started to be explored for effective removal of plastic contaminants from water. In particular, membrane bioreactors (MBRs) came out to be the most promising in microplastics removal reaching a removal efficiency of 99.99%. However, other membrane processes such as reverse osmosis (RO) and dynamic membranes technology are under investigation as possible alternative solutions for water remediation from plastics. 

The work from Nikolaeva and Luis [9] critically reviewed the separation performance of polymer-derived polyelectrolytes (PEs) membranes for the post-combustion CO_2_ capture. One of the main features of PEs is their charge that can be positive, negative or both. PEs polymer precursors are represented by uncharged polymers (such as chitosan), anionic polyelectrolites (such as sodium hyaluronate) and cationic polyelectrolites (such as poly(allylamine) hydrochloride). On the basis of the precursors employed, the PEs synthesis method changes (ionization, quaternisation, sulphation, cross-linking). The membranes are, then, prepared using different preparation techniques and including solvent casting, layer-by-layer (LbL) assembly, chemical grafting. The preparation technique greatly influences the CO_2_ separation performance of PEs membranes. Membranes prepared by the LbL technique display high CO_2_ selectivity but low CO_2_ flux. On the contrary, membranes prepared by solvent casting and chemical grafting exhibit high CO_2_ but limited selectivity. Many of the PEs membranes, thanks to their easy fabrication and high performance, can be suitable candidates for their exploitation at the industrial level for CO_2_ separation from flue gas. 

Monitoring membrane processes at the molecular level is one of the challenges that the scientific community is facing in order to deeply study and understand, at sub-micron scale, transport phenomena, fouling and biofouling onset and membrane behavior under different operating conditions.

The original article from Santoro et al. [10] aimed to monitor thermal polarization phenomenon in direct contact membrane distillation (DCMD) using electrospun nanofiber membranes (ENMs) in polyvinylidene fluoride (PVDF) loaded with a temperature-sensitive luminophore molecule ((Ru(phen)3) able to respond to local temperature changes. The immobilization of the luminophore allowed to determine, in a non-invasive way and directly at the membrane surface, the temperature polarization of the system giving fundamental hints for optimizing the process operating conditions.
